# Identification of a Novel Genotype of Severe Fever with Thrombocytopenia Syndrome Virus (SFTSV) in Northern Hebei Province, China

**DOI:** 10.3390/v17121534

**Published:** 2025-11-23

**Authors:** Minghao Geng, Xueqi Wang, Yamei Wei, Yan Li, Yanan Cai, Jiandong Li, Caixiao Jiang, Xinyang Zhang, Wentao Wu, Nana Guo, Guangyue Han, Xu Han, Tiezhu Liu, Qi Li, Shiwen Wang

**Affiliations:** 1 National Key Laboratory of Intelligent Tracking and Forecasting for Infectious Diseases, National Institute for Viral Disease Control and Prevention, Chinese Center for Disease Control and Prevention, Beijing 102206, China; gmhcdc@126.com (M.G.); ldong121@126.com (J.L.); 2 Institute for Viral Disease Prevention and Control, Hebei Provincial Center for Disease Prevention and Control, Shijiazhuang 050000, China; weiyamei2013@163.com (Y.W.); lian.2002@163.com (Y.L.); yanan589@163.com (Y.C.); jiangcaixiao@163.com (C.J.); zxhuade@126.com (X.Z.); wuwentao@zju.edu.cn (W.W.); yufeiwet@163.com (N.G.); hanguangyue2013@163.com (G.H.); hbcdchanxu@163.com (X.H.); 3 Capital Center for Children’s Health, Capital Medical University, Capital Institute of Pediatrics, Beijing 102206, China; iwangxueqi@gmail.com; 4 Hebei Key Laboratory of Pathogens and Epidemiology of Infectious Diseases, Hebei Provincial Center for Disease Control and Prevention, Shijiazhuang 050000, China

**Keywords:** SFTSV, tick-borne disease, BEAST, recombination

## Abstract

Severe fever with thrombocytopenia syndrome (SFTS), caused by SFTS virus (SFTSV), is an emerging tick-borne disease in East Asia. SFTS monitoring has been carried out since 2010 in mainland China, but no confirmed human cases or infected vectors had been reported from the northern regions of Hebei Province. We intensified surveillance in this area by collecting serum samples from patients with fever of unknown origin (FUO) and ticks from local habitats. Subsequently, all collected samples were screened for SFTSV by qRT-PCR. SFTSV RNA was detected in two patient sera from Chengde (2.2%). In six, positive ticks were detected among the *Haemaphysalis verticalis* (8.6%) collected from Zhangjiakou; no positive ticks were detected among the ticks collected from Chengde. Complete viral genomes were recovered from positive tick samples via next-generation sequencing and subjected to a suite of bioinformatic analyses. Two complete genomes from *Haemaphysalis verticalis* formed a distinct clade with an Inner Mongolia strain across L/M/S (bootstrap = 1.0) and separate from genotypes A–F; pairwise p-distances to genotypes A–F were >0.11 across L/M/S, supporting designation of a distinct genotype. We designate this lineage as genotype G; no credible recombination was detected. Based on the L segment, molecular-clock analyses dated the genotype G lineage to the late 13th century, predating the crown age of genotypes A–F (~18th century) by more than 400 years. We provide the first evidence of SFTSV circulation in northern Hebei and identify a novel, deeply divergent lineage. This finding confirms the co-circulation of distinct viral lineages (G and F) within the province and provides critical new insights into the virus’s diversity and evolutionary history. These results expand the known range and genetic diversity of SFTSV, underscoring the need for enhanced surveillance and ecological investigation in emerging regions. It is necessary to strengthen public health education, improve the early diagnosis and treatment ability of medical workers, and provide a scientific basis for targeted public health interventions.

## 1. Introduction

Severe Fever with Thrombocytopenia Syndrome (SFTS) is an emerging tick-borne infectious disease caused by the SFTS virus (SFTSV), a negative-sense RNA virus in the genus *Phlebovirus* of the family *Phenuiviridae* [1]. The SFTSV genome consists of three single-stranded, negative-sense RNA segments: Large (L), Medium (M), and Small (S), encoding the RNA-dependent RNA polymerase (RdRp), glycoproteins (Gp), and nucleocapsid protein (NP) and non-structural protein (NSs) [2]. Since its initial identification in Henan Province, China, in 2009 [1], SFTSV has posed an increasing threat to public health in East Asia, with expanding reports of human cases across China [3,4], South Korea [5], Japan [6], and Vietnam [7], posing a serious threat to public health. Clinically, SFTS is characterized by high fever, thrombocytopenia, leukopenia, gastrointestinal symptoms, and in severe cases, multi-organ dysfunction and death [8,9]. The virus is primarily transmitted via bites from infected ticks, particularly *Haemaphysalis longicornis* [10,11], and can also be transmitted through blood contact from infected animals or humans [12,13].

In recent years, the southern city of Cangzhou in Hebei Province has reported multiple confirmed SFTS cases [14]. By contrast, the northern regions of Hebei, such as Zhangjiakou and Chengde, which borders Beijing, had not previously reported any human SFTS cases or SFTSV-positive vectors. We therefore conducted molecular surveillance to assess previously undetected circulation, sampling sera from patients with fever of unknown origin (FUO) and field-collected ticks. Detection of SFTSV RNA in both human sera and ticks provides, to our knowledge, the first confirmation of viral activity in northern Hebei. Moreover, genome sequencing and phylogenetic analysis revealed a novel genotype distinct from the six recognized genotypes (A–F) [15]. These findings provide new insights into the genetic diversity, molecular evolution, and potential public health risks of SFTSV in northern Hebei.

## 2. Materials and Methods

### 2.1. Sample Collection

**Figure 1:**
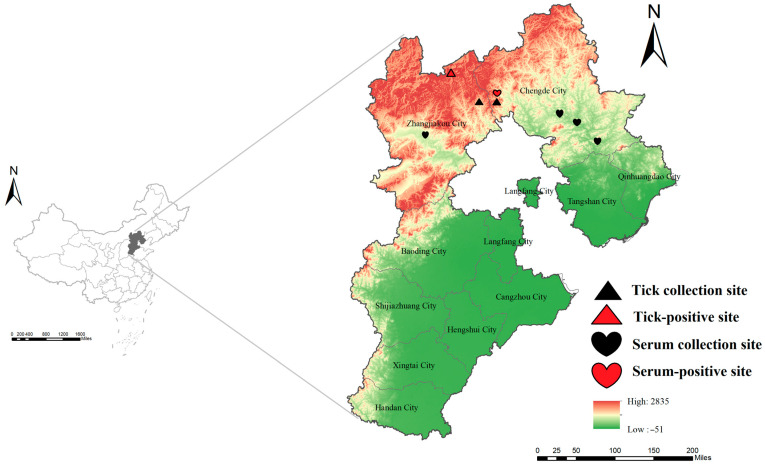
Map of Hebei Province in China. Map source: The Chinese Academy of Surveying and Mapping.

From 2017 to 2024, patients with FUO presenting to hospitals in Zhangjiakou and Chengde were enrolled as suspected SFTS cases and had serum collected. Concurrently, ticks were collected from forested, shrubby, and grassland habitats in surrounding rural areas using flagging and dragging methods in August 2024 (Figure 1). Captured ticks were morphologically identified to the species level by experienced entomologists. All samples were transported under cold chain conditions and stored at −80 °C until processing.

### 2.2. RNA Extraction and qRT-PCR Detection of SFTSV

Whole blood samples were collected in non-anticoagulant tubes. The samples were allowed to clot at room temperature for 3 h, followed by centrifugation at 3000× *g* for 10 min at 4 °C to separate the serum. The resulting serum was carefully aspirated, aliquoted, and immediately cryopreserved at −80 °C until further analysis. The collected ticks were first washed three times with phosphate-buffered saline (PBS) to remove surface contaminants. Each tick was individually homogenized in a separate tube with PBS at 4 °C using a Tissuelyzer II (Qiagen, Hilden, Germany). The homogenates were then centrifuged at 10,000× *g* for 10 min at 4 °C. The resulting supernatants were collected and subjected to nucleic acid extraction for downstream analyses.

Viral RNA was extracted from serum samples of patients with suspected SFTS, as well as from tick suspensions, using a nucleic acid isolation kit (Jiangsu Bioperfectus Technologies Co., Ltd., Taizhou, China) according to the manufacturer’s instructions. Real-time RT-PCR assays for SFTSV RNA detection were performed using a commercial diagnostic kit (Guangzhou Daan Gene Technology Co., Ltd., Guangzhou, China) following the manufacturer’s protocol.

### 2.3. Virus Isolation and Whole-Genome Sequencing

Tick homogenates that tested positive for SFTSV by qRT-PCR were inoculated onto Vero E6 cell monolayers to attempt virus isolation. After 5–7 days of incubation, culture supernatants were collected and screened for SFTSV RNA by qRT-PCR [16].

To obtain the complete viral genome, SFTSV-positive samples with a cycle threshold (Ct) value ≤ 30 were selected for sequencing. First-strand cDNA synthesis was performed using the SuperScript™ IV First-Strand Synthesis System (Thermo Fisher Scientific, Waltham, MA, USA) according to the manufacturer’s instructions, with the following cycling conditions: 25 °C for 10 min, 50 °C for 10 min, and 85 °C for 5 min. Previously published primer sets were used for the amplification of the S (three sets), M (seven sets), and L (twelve sets) segments (Table S1) [17]. PCR amplification was carried out on a thermal cycler under the following conditions: 94 °C for 30 s, 55 °C for 30 s, and 72 °C for 1 min, for a total of 35 cycles. Sequencing of the amplified products was performed on a NextSeq 2000 platform (Illumina, San Diego, CA, USA).

### 2.4. Phylogenetic and Evolutionary Analysis

The reference sequences was compiled from GenBank. These sequences were selected based on their genomic completeness (L, M, and S segments), ensuring they represented all six known genotypes (A–F) and originated from key endemic regions, including China, Japan, and the Republic of Korea, with collection dates spanning from 2010 to 2024.

Sequence editing was performed using the EditSeq module of the DNAStar software V11 suite to generate formatted sequences. Complete L, M, and S segment sequences were aligned using the ClustalW algorithm implemented in DNAStar [18]. Multiple sequence files were merged into a single Clustal format file using the SeqVerter program. Maximum-likelihood phylogenies were inferred in MEGA v11 [19] under the best-fit substitution model (GTR+G+I) selected by model testing, and node support was evaluated with 1000 bootstrap replicates. Pairwise distances were computed in MEGA v11 using the p-distance model with pairwise deletion of missing data; percent identity was calculated as 1 − p-distance.

### 2.5. Reassortment and Recombination

Potential recombination was screened with RDP v4.76 (RDP, GENECONV, Bootscan, MaxChi, Chimaera, SiScan, 3Seq). Events were considered credible only if detected by ≥3 methods with multiple-testing–corrected *p* < 0.05 [20]. For each strain, a recombination score was calculated by the software. Strains with a recombination score greater than 0.6 were regarded as highly credible recombinants, those with scores between 0.4 and 0.6 were considered putative recombinants, and strains with scores below 0.4 were considered unlikely to be recombinant [21]. Further validation was performed using the recombination analysis software SimPlot V3.5.1 [22]. The consistent results obtained from both RDP4 and SimPlot confirmed the accuracy and reliability of the recombination analysis. SimPlot software was also used to generate similarity plots using a sliding window approach (200 bp window, 20 bp step) to identify breakpoints and parental strains.

### 2.6. Phylogenetic Analyses by BEAST

The optimal nucleotide substitution model was determined using j ModelTest v2.1.7, which identified GTR+G+I as the best-fit model [23]. A molecular clock phylogeny of the L/M/S gene was reconstructed using BEAST v2.6.6 with a strict clock and the coalescent constant population size model [24]. Sampling dates were included to calibrate divergence times. Markov Chain Monte Carlo (MCMC) simulations were run for 200 million generations, with parameter settings adjusted to achieve parameter convergence and adequate effective sample sizes (ESS > 200), and results were summarized using TreeAnnotator with 10% burn-in [25,26]. The maximum clade credibility (MCC) tree was visualized in FigTree v1.4.3 software [27].

## 3. Results

### 3.1. Detection of SFTSV RNA in Patients and Ticks

From 2017 to 2024, we enrolled 92 patients with FUO in Hebei Province (Chengde *n* = 79; Zhangjiakou *n* = 13) and screened their sera for SFTSV RNA by qRT-PCR. Two patients from Chengde tested positive, yielding an overall positivity of 2.2% (2/92) and 2.5% in Chengde (2/79), with no positives in Zhangjiakou (0/13). The two positive cases were sampled in 2021 and 2024 and resided on the Bashang Plateau.

In August 2024, following the detection of SFTSV in human samples, we conducted tick surveillance in Chengde and Zhangjiakou. A total of 215 ticks were collected (Chengde, *n* = 60; Zhangjiakou, *n* = 155) and screened for SFTSV RNA by qRT-PCR. Species identification revealed that the 155 ticks collected from Zhangjiakou included 85 *Haemaphysalis longicornis* and 70 *Haemaphysalis verticalis*. In contrast, all 60 ticks collected from Chengde were identified as *Haemaphysalis longicornis*. SFTSV RNA was detected in six ticks from Zhangjiakou [3.9% (6/155)], with all six positive ticks identified as *Haemaphysalis verticalis* [8.6% (6/70)]. In contrast, no positives were found in Chengde [0% (0/60)], resulting in an overall tick positivity rate of 2.8% (6/215). Both cities lie on the Bashang Plateau and share similar geography, elevation, and climate. Taken together with the human detections, these data provide the first concurrent evidence of SFTSV in patients and ticks in northern Hebei.

### 3.2. Genomic Characteristics of SFTSV Isolates

Patient SFTSV-positive sera had high cycle threshold values (Ct > 33), indicating low viral loads. Virus isolation in cell culture was unsuccessful, and whole-genome sequencing did not recover complete or near-complete genomes, likely due to insufficient viral RNA.

Two SFTSV strains were successfully isolated from tick samples collected in Zhangjiakou, and their complete genomes, comprising the L (6368 nt), M (3378 nt), and S (1744 nt) segments, were obtained through next-generation sequencing (NGS). Pairwise comparison between the two genomes revealed high intra-lineage nucleotide identities of 99.55% (L), 98.36% (M), and 99.28% (S). Relative to Hebei genotype F strains (PQ827544), the new strains showed moderate nucleotide divergence but comparatively conserved proteins. For the L segment, nucleotide identity was 88.24–88.53%, whereas RdRp amino-acid identity was 97.36–97.41% (Tables S2 and S3), comprising 57 amino-acid substitutions distributed across multiple regions (PQ827544). For the M segment, nucleotide identity was 87.38–87.44% and GP amino-acid identity was 94.04–94.13% (Tables S4 and S5), with 65 amino-acid differences, the highest protein-level variability among the three segments. For the S segment, nucleotide identity was 89.20–89.55%; NSs showed 91.47% (Tables S6 and S7) amino-acid identity with 24 variable sites, whereas NP was more conserved, with 96.36% identity and 9 variable sites (Table 1). This pattern is consistent with extensive synonymous change under purifying selection.

### 3.3. Phylogenetic Analysis of SFTSV

**Figure 2:**
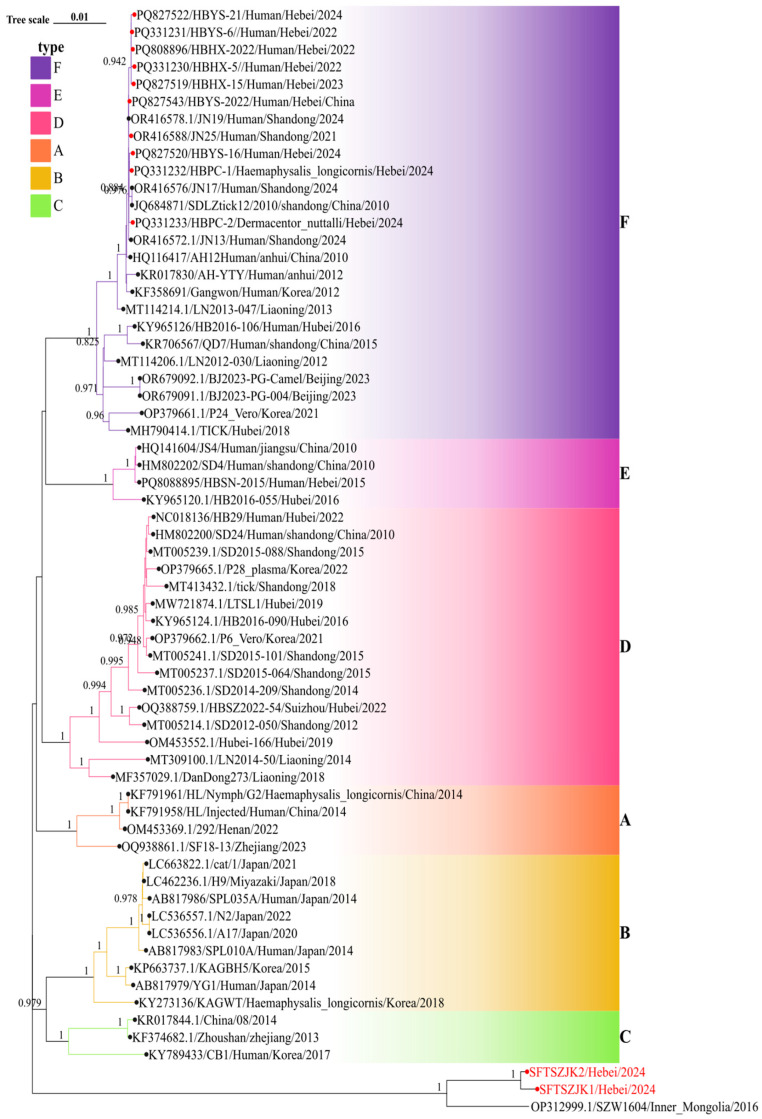
Phylogenetic relationships of SFTSV based on the L segments. Background colors indicate genotypes A–F. Red circles mark Hebei sequences; the red-labeled tips at the bottom are the two genomes from this study. In all three trees, these strains cluster with an Inner Mongolia strain as a distinct clade separate from genotypes A–F. Numbers at key nodes indicate bootstrap support; scale bar shows substitutions per site.

**Figure 3:**
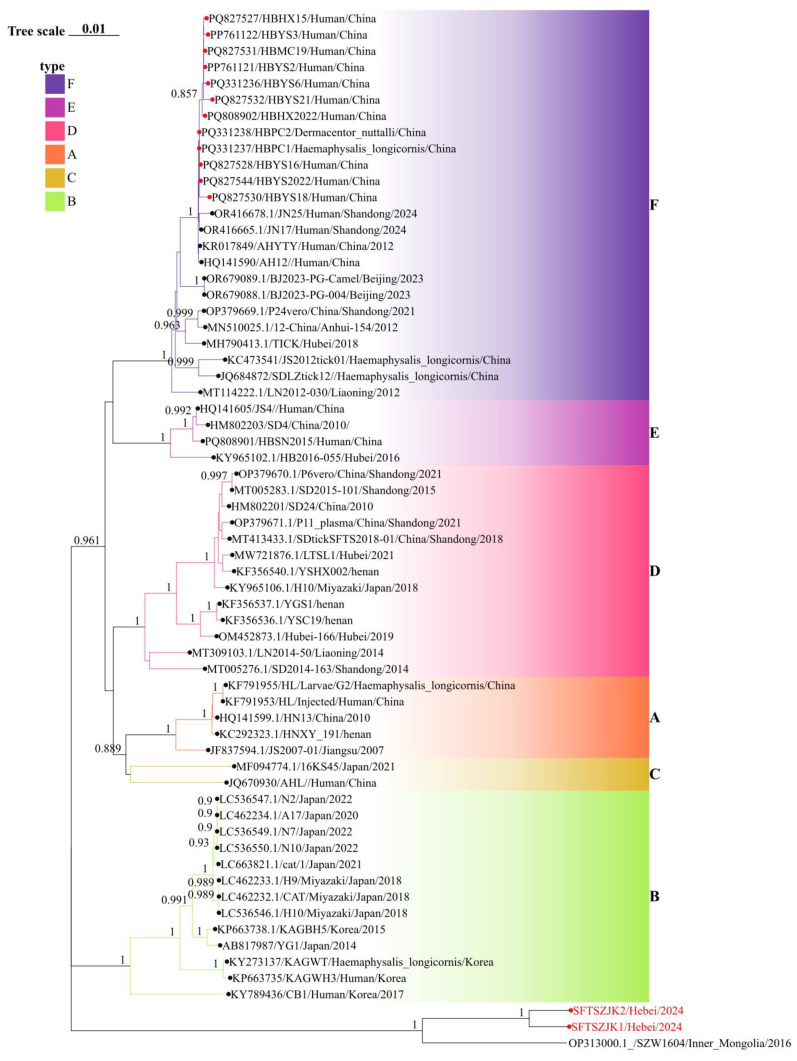
Phylogenetic relationships of SFTSV based on the M segments. Background colors indicate genotypes A–F. Red circles mark Hebei sequences; the red-labeled tips at the bottom are the two genomes from this study. In all three trees, these strains cluster with an Inner Mongolia strain as a distinct clade separate from genotypes A–F. Numbers at key nodes indicate bootstrap support; scale bar shows substitutions per site.

**Figure 4:**
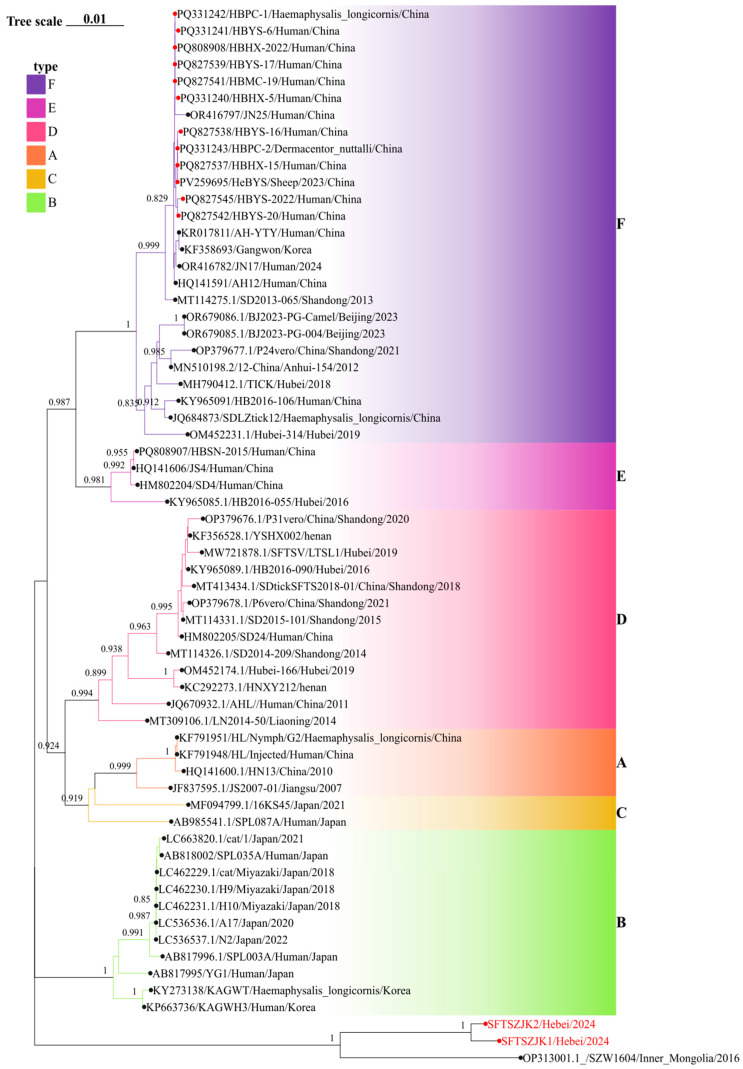
Phylogenetic relationships of SFTSV based on the S segments. Background colors indicate genotypes A–F. Red circles mark Hebei sequences; the red-labeled tips at the bottom are the two genomes from this study. In all three trees, these strains cluster with an Inner Mongolia strain as a distinct clade separate from genotypes A–F. Numbers at key nodes indicate bootstrap support; scale bar shows substitutions per site.

Phylogenetic trees were constructed for the complete L, M, and S segments of SFTSV using the maximum likelihood method with 1000 bootstrap replicates. All three trees consistently supported the clustering of the two novel sequences identified in this study (SFTSZJK1/Hebei/2024 and SFTSZJK2/Hebei/2024, highlighted in red, Figure 2, Figure 3 and Figure 4) into a distinct, well-supported clade (bootstrap = 1.0), clearly separated from the known genotypes A–F. The same topology was recovered for the L, M, and S segments (Figure 2). Our Hebei strains consistently clustered with a 2016 strain from Inner Mongolia (SZW1604/2016; OP312999.1), forming a distinct monophyletic clade. Pairwise genetic distances (p-distance) between the Hebei lineage and each of the six recognized genotypes (A–F) were >0.11 across L, M, and S, corresponding to ≤89% nucleotide identity. These values are higher than the typical inter-genotype distances observed among A–F, supporting the designation of a distinct genotype.

The congruent three segment placement and maximal bootstrap support justify proposing a novel SFTSV genotype, which we tentatively refer to as genotype G. Notably, the phylogenetic placement of our newly identified Genotype G strains stands in stark contrast to all previously characterized SFTSV isolates from Hebei Province. Prior surveillance efforts in Hebei have exclusively identified strains belonging to Genotype F, which were isolated from both human patients and ticks (marked as red circles in Figure 2, Figure 3 and Figure 4). These historical Hebei isolates show a close genetic relationship with strains circulating in the neighboring Shandong Province. The discovery of this divergent, ancient Genotype G lineage thus represents a paradigm shift in our understanding of SFTSV epidemiology in this region, revealing a previously unrecognized and distinct viral circulation.

### 3.4. Recombination Analysis

Potential recombination events were investigated using RDP4 (Table 2). The analysis identified one putative event in the L segment of SFTSZJK1/Hebei/2024 and another in the M segment of SFTSZJK2/Hebei/2024, whereas no recombination signals were detected in the S segment. Furthermore, the credibility of these signals was low, as evidenced by their low RDP recombination consensus scores (RDPRCS) of 0.446 and 0.436, and the fact that each was supported by only two of the seven detection algorithms (Table 2). Consistent with this weak evidence, a complementary analysis with SimPlot did not reveal any clear mosaic patterns indicative of recombination. Given that neither event met the criteria for a high-confidence signal, the genomes were treated as non-recombinant in subsequent phylogenetic analyses.

### 3.5. Evolutionary History and Divergence Time Estimation

**Figure 5:**
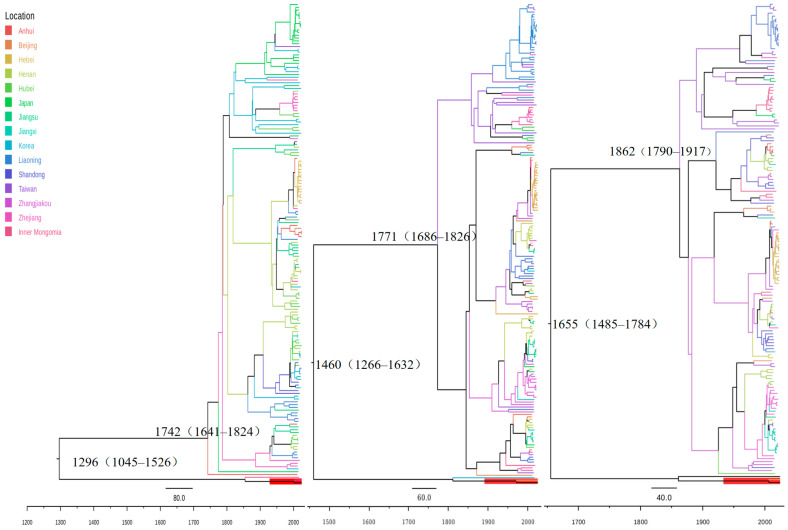
Time-scaled MCC trees of the L, M, and S segments of SFTSV. Branch colors indicate sampling locations. The red cluster at the bottom highlights the newly detected Hebei strains (SFTSZJK1/Hebei/2024, SFTSZJK2/Hebei/2024), which form a distinct clade. the time of most recent common ancestor (tMRCA) and 95%HPD time for each genotype and the posterior probability of major branches in the evolutionary tree were marked. The names of different colored strains in the evolutionary tree represent strains from different geographic locations.

To investigate the evolutionary timescale of the newly identified SFTSV lineage, we performed a Bayesian molecular clock analysis using BEAST. The SFTSV sequences from 2010 to 2024 utilized in this study were obtained directly from GenBank. The dataset comprised a total of 525 sequences (L: 171; M: 191; S: 162), which included sequences from major SFTSV endemic regions in China (such as Shandong, Henan, Anhui, and Hebei provinces) as well as from Japan and South Korea. The resulting maximum clade credibility (MCC) tree strongly supported the distinct phylogenetic position of our novel strains (SFTSZJK1/Hebei/2024 and SFTSZJK2/Hebei/2024) and the Inner Mongolia strain (OP312999.1), which formed an independent evolutionary clade with a posterior probability of 1 (Figure 5).

Time-scaled phylogenies inferred with BEAST yielded segment-specific evolutionary rates and divergence times for the L, M and S segment sequences. The mean substitution rate for the L segment was 1.02 × 10^−4^ substitutions/site/year (95% HPD, 6.78 × 10^−5^–1.33 × 10^−4^), for the M segment 1.51 × 10^−4^ substitutions/site/year (95% HPD, 1.05 × 10^−4^–1.95 × 10^−4^), and for the S segment 1.85 × 10^−4^ substitutions/site/year (95% HPD, 1.18 × 10^−4^–2.55 × 10^−4^). Median divergence times of the new lineage from all other SFTSV lineages were estimated at 1296 (95% HPD: 1045–1526) CE for L, 1475 (95% HPD: 1266–1632) CE for M, and 1655 (95% HPD: 1485–1784) CE for S. Despite differences among segments, all estimates place the split several centuries ago. The time-scaled phylogeny revealed a striking temporal divergence for the novel Genotype G. While the time to the most recent common ancestor (tMRCA) for all previously known the L segment of SFTSV genotypes (A–F) was estimated to the mid-18th century [28], the tMRCA for Genotype G was dated profoundly earlier to approximately the year 1296 (95% HPD: 1045–1526). This finding establishes that Genotype G is not a recent offshoot of the known SFTSV radiation but is instead a deeply diverged, ancient lineage that predates the common ancestor of all other known strains by over 450 years, indicating a long-term, regionally maintained evolutionary history in northern Hebei.

## 4. Discussion

Severe Fever with Thrombocytopenia Syndrome Virus (SFTSV) has emerged as a significant public health threat across East Asia since its discovery in 2009 [1]. After 2022, the number of SFTS cases in Hebei Province has gradually increased [14], while its northern territory adjoins the Inner Mongolia Plateau. Despite extensive surveillance, the vast northern regions of Hebei Province, including the cities of Chengde and Zhangjiakou, with no previously reported human cases or viral presence in vectors.

In this study, our investigation was initially prompted by the detection of SFTSV RNA in two serum samples during a retrospective screening of FUO patients in northern Hebei Province. Although whole-genome sequencing of these human samples was unsuccessful due to high Ct values indicative of low viral loads, this finding provided the first crucial evidence of potential SFTSV infections in the local population. This prompted us to conduct an immediate entomological follow-up survey to assess the public health risk. We collected 215 ticks from Zhangjioukou and Chengde cities, of which six tested positive for SFTSV RNA. Subsequently, using next-generation sequencing on these positive tick samples, we successfully recovered two complete viral genomes. To our knowledge, this study represents the first concurrent detection of SFTSV in both human patients and tick vectors in northern Hebei Province, confirming the existence of active local transmission cycles and highlighting a previously unrecognized public health threat in this region.

Genome sequencing of SFTSV-positive ticks from Zhangjiakou yielded two complete genomes. Genetic distance analyses further reinforced the distinctiveness of the Hebei lineage. Using MEGA (p-distance; pairwise deletion) on the L/M/S alignment, the minimum pairwise distance between any Hebei strain and each of the six recognized genotypes (A–F) exceeded 0.11 (≤89% nucleotide identity). By contrast, between-group distances among genotypes A–F in the same dataset were substantially lower (0.01–0.04). This places the Hebei lineage well beyond the typical inter-genotype divergence observed within SFTSV. At the protein level, identities remained high (≈94–97% across RdRp, GP, NSs, and NP), consistent with predominantly synonymous changes and strong purifying selection on protein function. Maximum-likelihood phylogenies of the L, M, and S segments placed both sequences in a distinct, well-supported monophyletic clade with an Inner Mongolia strain [29] (SZW1604/2016) and clearly separated from genotypes A–F (bootstrap = 1). We provisionally designate this deeply diverged lineage as genotype G. Collectively, these data confirm genotype G as a phylogenetically independent lineage and represent a pivotal advance in defining SFTSV diversity, evolution, and geographic expansion in northern Hebei.

A pivotal implication of this study is a substantive revision of the SFTSV genetic landscape in Hebei Province. To date, all publicly reported Hebei isolates have clustered within genotype F [14] and are closely related to strains prevalent in neighboring Shandong [30]. The identification of a highly divergent lineage fundamentally alters this view and indicates the co-circulation of at least two genetically distinct SFTSV lineages within Hebei. We infer that genotype F represents the previously recognized transmission system linked to the North China Plain, whereas genotype G likely reflects a cryptic, enzootic cycle on the Bashang Plateau and Inner Mongolia Plateau. This dichotomy is consistent with putative vector partitioning, genotype G was detected in *Haemaphysalis verticalis*, whereas historical detections of genotype F in Hebei have involved *H. longicornis*, although sample sizes are limited and vector competence remains to be demonstrated.

The discovery of Genotype G in northern Hebei expands SFTSV’s known geographic range, which previously focused on central and eastern Chinese provinces [31,32,33]. The Bashang Plateau and Inner Mongolia Plateau, a region including Hebei, Inner Mongolia, and Shanxi, emerges as a plausible focus for this lineage. Ecological continuity across this area, characterized by similar elevation, climate, and vegetation, is likely accompanied by similarities in tick species and the viral strains they harbor, which facilitates viral transmission and spread. Vigilance should be heightened, and viral surveillance in this region should be strengthened. The close phylogenetic relationship between Genotype G strains and a 2016 Inner Mongolia isolate (SZW1604/2016) supports this regional transmission network. Although sample sizes are limited, these findings suggest ecological structuring of SFTSV transmission across northern versus southern Hebei.

While RDP4 identified some minor signals, the lack of consensus among multiple algorithms, the low recombination scores, and the absence of a clear recombinant pattern in the SimPlot analysis strongly suggest that recombination was not a major evolutionary mechanism in the formation of this novel genotype. Together, these data indicate that genotype G most likely arose through long-term divergent evolution rather than recent recombination. while acknowledging that ancient or rare events cannot be entirely excluded.

BEAST analyses consistently placed the two Hebei genomes with the Inner Mongolia strain in a maximally supported, independent clade, indicating a distinct lineage circulating in northern Hebei. Segment-specific clocks dated its split from all other SFTSV lineages to the Late Medieval to Early Modern Period (ca. 1296–1655), markedly earlier than the crown age of the A–F lineages (~mid-18th century) [34,35]. This finding marks a major shift in our understanding of the evolutionary mechanisms of SFTSV, indicating that its origin is far older and more complex than previously recognized, likely maintained by enzootic cycles involving local ticks and wildlife reservoirs [36]. Genotype G appears to be a unique genotype deeply rooted in the Bashang Plateau and Inner Mongolia Plateau, which connects northern Hebei Province with the Inner Mongolia Autonomous Region, and may have been quietly spreading in this region for centuries. The different rates (L: 1.02 × 10^−4^; M: 1.51 × 10^−4^; S: 1.85 × 10^−4^ substitutions/site/year) and divergence times across L/M/S are expected and informative for segmented bunyaviruses [35,37,38]. The RdRp-encoding L segment evolves slowest under strong purifying constraint, whereas the glycoprotein precursor coding M segment and the -coding S segment evolve faster due to host-receptor interaction and immune antagonism [39], respectively; the S-segment average likely reflects a mix of highly conserved NP and more labile NSs [26,28]. Additional contributors include unequal temporal and geographic sampling per segment, lineage-specific rate variation under a strict clock, and independent segment genealogies; thus point estimates need not coincide even without detectable reassortment. Importantly, the three segments are topologically congruent and all place genotype G’s origin centuries in the past, reinforcing its antiquity and regional distinctness. These findings have practical implications for public health in northern Hebei. Genotype-inclusive molecular surveillance should be expanded in Zhangjiakou, Chengde, and adjacent Inner Mongolia, integrating ticks, wildlife, and livestock. Diagnostic capacity should be strengthened by re-evaluating commonly used qRT-PCR primers and probes against genotype G and, where necessary, updating assays to ensure sensitivity across genotypes. In parallel, antigenic assessments are needed to gauge potential effects on serological detection and the breadth of candidate vaccines.

These findings have immediate public health implications for northern Hebei and adjacent areas, including the Beijing metropolitan region. Clinicians should consider SFTS in the differential diagnosis of acute febrile illness, particularly among patients with recent outdoor exposure. Public health agencies should recognize a newly identified endemic focus and implement a comprehensive, multi-pronged strategy for risk mitigation and control. This strategy necessitates immediate and widespread medical training programs to enhance the competency of local medical workers in recognizing the clinical and epidemiological presentation of SFTS. Such training is crucial for improving the rate and accuracy of early diagnosis, particularly given the non-specific early symptoms of the disease. Furthermore, targeted surveillance must be implemented, including routine monitoring of tick populations with emphasis on *Haemaphysalis verticalis*, sentinel surveillance in humans, livestock, and small mammals, and sustained spatiotemporal sampling across seasons [34,40]. Targeted interventions must also be prioritized, focusing on vector control in high-risk areas. Risk communication should be updated to emphasize tick-bite prevention for residents and visitors to the Bashang Plateau. Finally, diagnostic capacity should be strengthened by periodic in silico re-evaluation of commonly used qRT-PCR primers and probes against Genotype G, with assay updates as needed to maintain sensitivity across genotypes, and verification by laboratory testing and external quality assessment where feasible.

As surveillance expands, additional SFTS cases and divergent SFTSV genotypes are likely to be detected. From a public health perspective, our findings underscore the need to expand surveillance for SFTSV in non-endemic regions, particularly where ecological conditions resemble known endemic zones. Public awareness, physician training, and vector control should be prioritized in Zhangjiakou and Chengde to mitigate outbreak risk. We emphasize that this significant molecular divergence warrants immediate investigation into its virulence profile, specifically comparing Genotype G against circulating Genotype F strains in animal models to determine if the changes in RdRp and other structural proteins translate into altered pathogenicity, transmissibility, or clinical outcomes. If necessary, antigenic assessment targeting Genotype G should be conducted to evaluate its potential impacts on serological detection and the protective scope of candidate vaccines.

## 5. Conclusions

In conclusion, e document the first evidence of SFTSV circulation in northern Hebei and identify a novel, deeply diverged lineage (provisionally genotype G). Two complete genomes from *Haemaphysalis verticalis* formed a three-segment–concordant clade with an Inner Mongolia strain, distinct from genotypes A–F and without evidence of recent recombination; molecular-clock analyses date the L segment of this lineage to the late 13th century, indicating long-term regional maintenance. These results expand the known range and genetic diversity of SFTSV and underscore the need for heightened surveillance and ecological investigation in emerging regions. Strengthened detection and control measures in Zhangjiakou and Chengde are critical for anticipating future outbreaks and understanding the evolutionary trajectory of SFTSV.

## Figures and Tables

**Table 1:** Sequence information of isolated SFTSV strains.

SFTSV Strain	Source	GenBank Accession No. (L/M/S)	Sequence Similarity (%)						
			L Segment		M Segment		S Segment		
			Nt	RDRP	Nt	GP	Nt	NSs	NP
SFTSZJK1/Hebei/2024	*Haemaphysalis verticalis*	PX226566/PX226564/ PX226562	88.53	97.36	87.44	94.13	89.20	91.47	96.36
SFTSZJK2/Hebei/2024	*Haemaphysalis verticalis*	PX226567/PX226565/ PX226563	88.24	97.41	87.38	94.04	89.55	91.47	96.36

**Table 2:** Recombination statistics for L and M SFTSV segments in Hebei province.

Gene	L	M
Recombinant Sequence(s)	SFTSZJK1/Hebei/2024	SFTSZJK2/Hebei/2024
Beginning	66	1607
Ending	6322	1730
RDPRCS	0.446	0.436
RDP	8.39 × 10^−6^	4.02 × 10^−4^
GENECONV	NS	NS
Bootscan	NS	NS
Maxchi	NS	1.42 × 10^−4^
Chimaera	NS	NS
SiSscan	NS	NS
3Seq	5.50 × 10^−4^	NS

## Data Availability

All the sequences have been submitted to the Genbank.

## Supplementary Materials

The following supporting information can be downloaded at: https://www.mdpi.com/article/10.3390/v17121534/s1, Table S1. The specific primer sets used for whole SFTSV genome amplification; Table S2. L fragment nucleotide similarity; Table S3. RdRp amino acid variation sites; Table S4. M fragment nucleotide similarity; Table S5. Gp amino acid variation sites. Table S6. S fragment nucleotide similarity; Table S7. NSs amino acid variation sites.
