# Correction: Geng et al. Identification of a Novel Genotype of Severe Fever with Thrombocytopenia Syndrome Virus (SFTSV) in Northern Hebei Province, China. *Viruses* 2025, *17*, 1534

**DOI:** 10.3390/v18010114

**Published:** 2026-01-15

**Authors:** Minghao Geng, Xueqi Wang, Yamei Wei, Yan Li, Yanan Cai, Jiandong Li, Caixiao Jiang, Xinyang Zhang, Wentao Wu, Nana Guo, Guangyue Han, Xu Han, Tiezhu Liu, Qi Li, Shiwen Wang

**Affiliations:** 1 National Key Laboratory of Intelligent Tracking and Forecasting for Infectious Diseases, National Institute for Viral Disease Control and Prevention, Chinese Center for Disease Control and Prevention, Beijing 102206, China; gmhcdc@126.com (M.G.); ldong121@126.com (J.L.); 2 Institute for Viral Disease Prevention and Control, Hebei Provincial Center for Disease Prevention and Control, Shijiazhuang 050000, China; weiyamei2013@163.com (Y.W.); lian.2002@163.com (Y.L.); yanan589@163.com (Y.C.); jiangcaixiao@163.com (C.J.); zxhuade@126.com (X.Z.); wuwentao@zju.edu.cn (W.W.); yufeiwet@163.com (N.G.); hanguangyue2013@163.com (G.H.); hbcdchanxu@163.com (X.H.); 3 Capital Center for Children’s Health, Capital Medical University, Capital Institute of Pediatrics, Beijing 102206, China; iwangxueqi@gmail.com; 4 Hebei Key Laboratory of Pathogens and Epidemiology of Infectious Diseases, Hebei Provincial Center for Disease Control and Prevention, Shijiazhuang 050000, China

## Error in Figure 3

In the original publication [1], there was a mistake in Figure 3 as published. Figure 3 was inadvertently submitted as the same image as Figure 2. The corrected Figure 3 appears below.

The authors state that the scientific conclusions are unaffected. This correction was approved by the Academic Editor. The original publication has also been updated.

## Figures and Tables

**Figure 3:**
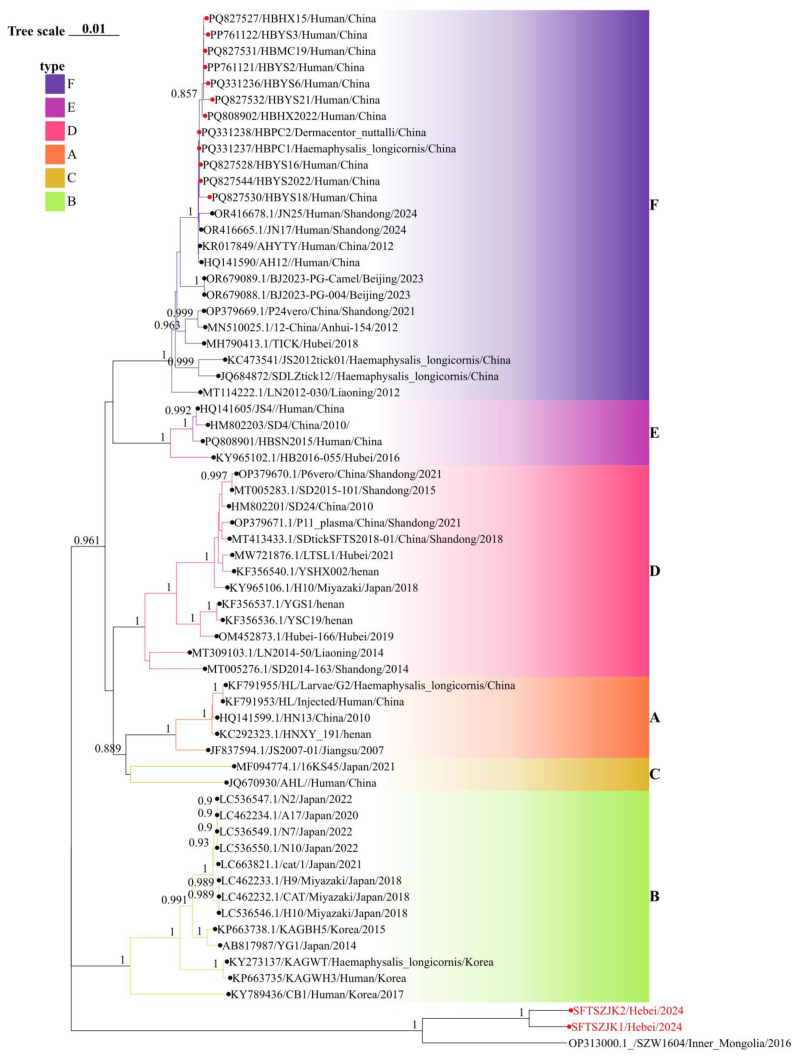
Phylogenetic relationships of SFTSV based on the M segments. Background colors indicate genotypes A–F. Red circles mark Hebei sequences; the red-labeled tips at the bottom are the two genomes from this study. In all three trees, these strains cluster with an Inner Mongolia strain as a distinct clade separate from genotypes A–F. Numbers at key nodes indicate bootstrap support; scale bar shows substitutions per site.
