# Supplementary material for: Identification of a Novel Genotype of Severe Fever with Thrombocytopenia Syndrome Virus (SFTSV) in Northern Hebei Province, China
Source: Viruses. 2025 Nov 23;17(12):1534. doi: 10.3390/v17121534 (PMC12737708; doi:10.3390/v17121534)
Supplement: Supplementary file 1 [file viruses-17-01534-s001.zip › Table S1. The specific primer sets used for whole SFTSV genome amplification.pdf]

Table S1. The specific primer sets used for whole SFTSV genome amplification.

Segment	Forward Name	Forward Sequence	Reverse Name	Reverse Sequence	Size (bp)
L	LF-1F	ACACAGAGACGCCAGATG	LF-1R	GAACCCCTCCTGACGAGACTAC	548
	LF-2F	CAACCACTAGGAGCCATAA	LF-2R	TGTCCCTCGATTCAATGATGT	605
	LF-3F	ATCACCAAGACCCTCAAAGC	LF-3R	TCCAACAAGATTGACACAGCTC	668
	LF-4F	GCAGCAAACCAGAAAGAAAGA	LF-4R	CTCCATCTGGGTGTACCTGT	706
	LF-5F	AGAGAGAAGTTGGGCGTGGC	LF-5R	AGGATGTTCTTGGCCAATCTC	660
	LF-6F	AGCTTCCTCAGAGCTGCTTG	LF-6R	CCTTAGGTCCACCATCATCTT	684
	LF-7F	GCCAAGAAGTGGAATCAGG	LF-7R	ATGAGGGCCCCTCCAAGC	739
	LF-8F	TTCCACAGGCACTTGGTTTAG	LF-8R	CGAAAGCAGTGGGCGTGAGAG	707
	LF-9F	GCTGCCCAACAAGAAGGAAC	LF-9R	TCTAGGCTAAAACCAGGGA	710
	LF-10F	TTAGGAACTTCATAGCCCACG	LF-10R	CCGTCAGTCCTTGATGCTGG	709
	LF-11F	GTAGTGATGCCAGGCTTTATG	LF-11R	CTTCTCCAAACTCTTCCACCTC	738
	LF-12F	GGTCAAAGCTCACAGAGATGG	LF-12R	AGACCGCCCAGATCTTAAGGAAATC	546
M	MF-1F	ACACAGAGACGGCCAACAATG	MF-1R	TCTCCTCAGGGATGGGTGTCA	586
	MF-2F	GATAGTTCCTGGGCCTTCATACAA	MF-2R	GAACCRTAGCACTTTGGTCTGA	640
	MF-3F	GAGGCATCTGAGGCCAAGTG	MF-3R	CCTATTTCCTCATGGATCACTTGC	677
	MF-4F	TGGGGTCATGGGTCATAGCTC	MF-4R	CTGCCCAATCATCAGAAAAGG	586
	MF-5F	GGAGTCCGGACTCAAAATGTC	MF-5R	GCGTCATCCACYCGTAGCTC	685
	MF-6F	TTAGCTCCCTGCAACCAGGC	MF-6R	CCACAAATTTGGGACATCCAG	634
	MF-7F	GGGATGAGACTGCATTCAGTG	MF-7R	ACACAAAGACCGGCCAACACTTC	527
S	SF-1F	ACACAAAGACCCCCTTCATTTGG	SF-1R	GCTCATCATCCTCATCCAAGACAC	749
	SF-2F	CTCCTCCAGATAGAGTCACTTGCA	SF-2R	TGAAGCCACAACCAAGACCCT	646
	SF-3F	TAAACTTCTGTTCTGCTGGCTCC	SF-3R	ACACAAAGACCCCCAAAAAAGG	622
